# How to Design the Control Group in Randomized Controlled Trials of Acupuncture?

**DOI:** 10.1155/2012/875284

**Published:** 2012-07-05

**Authors:** Jaung-Geng Lin, Chao-Hsun Chen, Yu-Che Huang, Yi-Hung Chen

**Affiliations:** ^1^School of Chinese Medicine, China Medical University, No. 91 Hsueh-Shih Road, Taichung 40402, Taiwan; ^2^Chang-Hua Hospital Department of Health Executive Yuan, No. 80, Sec. 2, Zhongzheng Road, Puxin Township, Changhua County 51341, Taiwan; ^3^Graduate Institute of Acupuncture Science, China Medical University, No. 91 Hsueh-Shih Road, Taichung 40402, Taiwan

## Abstract

In evidence-based medicine, randomized controlled trials (RCTs) are the preferred method for evaluating the efficacy of interventions. In regard to acupuncture RCTs, the most difficult issues are the design of the control group and implementation of the principle of “double-blinding.” We compared the advantages and limitations associated with different control group designs in acupuncture RCTs, to assist researchers in this field.

## 1. Introduction

### 1.1. The WHO Promotes the Use and Research of Acupuncture

Acupuncture is a convenient, effective, and simple form of traditional Chinese medicine that has few side effects [1]. Thus, it is often the first therapy that patients consider for a wide range of complaints where medicine is lacking and is the most widely used complementary and alternative medicine worldwide. In 1979, the World Health Organization (WHO) issued its first report on traditional medicine and described 43 categories of symptoms treatable by acupuncture; in 1996, the list was expanded to 64 [2]. This fact alone shows the value of acupuncture worldwide. In 1995, the WHO “Guidelines for Clinical Research on Acupuncture” [3] stipulated three criteria for good acupuncture studies: validity, reliability, and statistical significance (*P* value). Thus, at every stage of research, from basic studies to clinical trials, these criteria are paramount in any attempt to explain the function and effect of acupuncture.

### 1.2. Acupuncture RCTs Are Needed

Evidence-based medicine (EBM) [4] has become the gold standard among researchers, ever since the term was coined by Guyatt et al. in his paper published in *JAMA* in 1992 [5]. Randomized clinical trials (RCTs) belong to the Ib [6] level of EBM and therefore provide reliable criteria for study validity. Factors influencing the validity of RCTs include [3] patient selection, study size, site(s) of investigation, blinding techniques, randomization, design of the control group, crossover studies (usually inappropriate for acupuncture), and the strategic approach. Well-designed, properly conducted RCTs also include statistical analysis by a biostatistician to ensure that the results are statistically valid.

### 1.3. The Importance of a Control Group in Acupuncture RCTs

The ongoing debate about the therapeutic effects of acupuncture for some diseases [7–12] means that RCTs are needed to evaluate the efficacy of acupuncture. Such trials must include an appropriate control group [13]. A well-designed control group not only increases reliability of the study but also improves its blinding, which further affects the study results [14].

One researcher [15] has questioned the consistently positive clinical trials of acupuncture from countries in East Asia. His systematic review of controlled trials noted that six studies had insufficient blinding. Inadequate concealment from the control group and lack of randomization yielded a higher ratio of positive results in these studies. Furthermore, any mistakes in selection or having a poorly designed control group may lead to misinterpretation of results or misleading statements [16].

The purpose of this study was to systematically review the construction of control groups in RCT acupuncture studies published over the last few decades.

## 2. Methods

A literature search was performed using the PubMed English language database, using the search keywords “acupuncture” and “placebo needle.” We included studies that met the following criteria: randomized control trials (RCTs) that adopted a double-blind, single-blind, or nonblind design. We chose studies that we considered to be representative for each category of control group design. We focus our analysis on the design of control groups in acupuncture studies, instead of evaluating the efficacy of acupuncture in each study.

## 3. Results

### 3.1. The Types, Advantages, and Limitations of Control Groups in Acupuncture RCTs

A survey of the literature reveals that 11 different designs exist for control groups in acupuncture RCTs, as shown in Table 1. Among these 11 designs, those classified under 1–5 have no needle intervention, those under 6–8 vary from the treatment group according to the location and depth of needle insertion, those under 9-10 use assistant tools on the skin surface, while the last design comprises multiple methods using paired subjects/controls. Advantages and limitations associated with each type are listed in Table 1.

## 4. Discussion

### 4.1. The Ideal Control Group

The purpose of the control group is to determine the effect of the intervention by properly eliminating any placebo effect produced by the test group. Therefore, RCT study designs must include at least 1 control group. Importantly, the control group must experience the same placebo effect as the test group, and ideally participants and researchers are blinded as to whether participants receive treatment or placebo.

An ideal acupuncture control group must include three conditions. First, the design should be accepted by the study subjects, who must be blinded as to their assigned treatment group. Second, the curative effects should be minor and not give rise to special therapeutic effects that influence the results. Third, all other conditions for controls, excluding the intervention, should be identical to those used for the test group.

### 4.2. Design Strategy

Four different strategies for designing control groups have been used alone or in combination in recent acupuncture RCTs: (1) absence of acupuncture needle insertion, (2) different location of inserted acupuncture needles, (3) different depth of insertion, and (4) the use of assistant tools. In regard to the absence of acupuncture needle insertion, the control group employed an imitative puncture action and no acupuncture, performed at specific acupoints, nonspecific acupoints, or sham acupoints. In regard to different locations for inserted acupuncture needles, specific acupoints consisted of the acupoints applied in the test group according to the meridian method of acupuncture, while sham acupoints comprised the points drifting off the specific acupoints or meridian above 0.5 B-cun and not on the meridian or known acupoints. However, the optimal distance for drifting off the specific acupoints or meridian remains controversial. Nonspecific acupoints are acupoints with nontherapeutic effects or minor effects that have been observed in previous research. In regard to different depth of insertion, needle insertion depths are categorized as superficial, minimal, or deep acupuncture. Superficial acupuncture does not pierce the epidermis. In minimal acupuncture, the needle penetrates to a depth of less than 4 mm and does not cause “de-chi.” Deep acupuncture involves needle passage through the hypodermis to a depth of more than 5 mm, usually 10–20 mm, and often causes “de-chi.” In reference to the use of assistant tools, placebo needles [17–20] such as the Streitberger's needle, Park Sham Device, or Takakura's needle use a blunt tip and are tapped onto the skin. It is important to note that, although these four strategies were adopted, the control group in acupuncture RCTs is less of an ideal comparator than when it is used in drug RCTs.

### 4.3. Laser Acupuncture and Transcutaneous Electrical Nerve Stimulation (TENS)

Laser acupuncture and TENS usually serve as nonacupuncture contrasts, meaning that they are not involved in needle insertion. Laser acupuncture has been clinically applied since the 1970s [21]. Laser acupuncture is defined as the simulation of traditional acupuncture points with low-intensity, nonthermal laser irradiation. The therapeutic effects are related to not only wavelength, irradiance, and beam profile, but also skin properties such as thickness, age, and pigmentation [21]. Laser acupuncture may be beneficial for conditions ranging from pain relief, hiccups to enuresis, and postoperative nausea and vomiting [22, 23]. These possible therapeutic effects may mean that using laser acupuncture as an intervention in the control group may cause underestimation of acupuncture effects.

TENS is a noninvasive analgesic technique that is usually used to relieve nociceptive, neuropathic, and musculoskeletal pain [24]. Evidence from animal studies has shown that TENS reduces ongoing nociceptive cell activity and sensitization in the central nervous system when applied to somatic receptive fields. It is hypothesized that low-intensity TENS relieves pain by a segmental mechanism, while higher intensity TENS activates extrasegmental descending pain inhibitory pathways [25].

Because of these possible therapeutic effects, using TENS as the control group intervention may cause underestimation of acupuncture effects. Moreover, the procedure of TENS differs from acupuncture. Therefore, blinding is impossible. It is noted that TENS should not be applied on the anterior and posterior areas of the chest because TENS may compromise pulmonary ventilation due to the stimulation of the intercostal muscles [25]. TENS should not be placed over areas where there has been recent haemorrhage because the currents are able to cause further haemorrhage.

### 4.4. Needle Pricking May Induce Nonspecific Physiological Reactions

Needle pricking may induce nonspecific physiological reactions and diffuse noxious inhibitory controls (DNICs) [26]. When a nociceptive stimulus is applied to any part of the body that is distinct from the excitatory receptive fields, discharges of neurons in the dorsal horn of the spinal cord are strongly inhibited. Sham needles penetration into sites other than acupoints or nonspecific needles into nontherapeutic acupoints n may act as nociceptive stimuli. These stimuli then activate the A delta and/or peripheral C-fibers, triggering DNIC [27]. Hence, in pain research, acupuncture may decrease pain sensation among subjects in the control groups using sham and nonspecific contrast designs and thereby reduce any between-group differences. Therefore, we suggest that acupuncture analgesia RCTs adopt minimal acupuncture contrast, superficial acupuncture contrast, or a placebo needle contrast design for the control group.

### 4.5. Minimal Acupuncture and Superficial Acupuncture

Minimal acupuncture requires that insertion points are not acupoints and needle penetration is to a depth of less than 4 mm, while superficial acupuncture requires that dull needles or other tools (e.g., needle tube and toothpicks) are tapped onto acupoints; dull needles do not prick the skin. It was assumed that both interventions have no therapeutic effects. However, recent studies did not agree with this assumption. Lightly touching the skin stimulates mechanoreceptors coupled to slow conducting unmyelinated C afferents that causes activity in the insular region, but not in the somatosensory cortex [28, 29]. This afferent nerve activity may affect brain function resulting in a “limbic touch response,” which causes emotional and hormonal reactions. It is likely that, in many studies, when minimal acupuncture and superficial acupuncture are used as control interventions, they are able to alleviate the pain condition. However, compared to nonspecific and sham acupuncture involving real needle insertion, minimal acupuncture and superficial acupuncture are considered to have less influence upon levels of pain.

### 4.6. Nonspecific and Sham Acupuncture

Nonspecific acupuncture involves acupuncture performed at acupoints that are considered to produce only minor or no effects. However, care must be taken as to choice of nonspecific acupoints: inappropriate selection will render the study invalid. Not only does it remain controversial as to the optimal distance for drifting off specific acupoints or meridian when using sham acupuncture, but also, if two meridians are sited closely together, the sham acupoints may be located on an unintended meridian. For long-term study, both nonspecific and sham acupuncture are considered to be more appropriate as control interventions because they involve real needle insertion.

### 4.7. Placebo Needling

Placebo needling is considered to be a credible technique for use in subjects with little or no experience of acupuncture [17–20]. However, studies have found that placebo needling had a greater placebo effect on subjects than did placebo pills [30, 31]. The more knowledge and experience among study participants have as to acupuncture the deeper the controversy. Moreover, the therapeutic procedure may produce a psychological placebo effect, instead of real physiological phenomena that are associated with true acupuncture. Selection of acupuncture points and the visual impact of needling also influence the applicability of placebo needling [32]. More importantly, those devices (the placebo needle) do not overcome the problem of double-blinding [33]. However, the blinding effect is increased when placebo needles are used together with needle tubes [34–39]. While Takakura's placebo needle blinds the acupuncturist to a greater degree compared with the Park Sham Device (PSD) and Streitberger's needles, the blinding is less effective for participants, because the needle sensation is less than that produced by the PSD and Streitberger's needles.

## 5. Conclusions

We list 11 different designs and four strategies associated with control group design in acupuncture RCTs. In clinical practice experience, efficacy is closely related to the manipulation of acupuncture performance, including the lifting and thrusting of needles or needle rotation. For example, in acupuncture analgesia, stronger stimulation (rotating needles with higher frequency and a thicker needle) will have greater efficacy than a weaker stimulation (rotating needles with lower frequency and with a thinner needle). Acupuncture manipulation is not easy to describe very clearly, so most acupuncture RCTs are performed with electroacupuncture. We suggest that acupuncture RCTs may be successfully conducted by hand manipulation if the method is properly described, including details such as the reinforcing and reducing method and acupuncture dosage. In this way, acupuncture research will be more closely related to clinical practice.

Hence, the choice of control in an RCT depends on the type of research and the therapeutic effects of acupuncture being examined. The research design should take into consideration the placebo effect, a blinded design, subject selection, measurements chosen, and any contrast between the treatment and placebo groups. Such a design will correctly reflect the result without underestimating the effect of acupuncture.

## Figures and Tables

**Table 1:** Acupuncture study control group designs.

Number	Categories	Method	Purpose	Advantage	Limitation
(1)	Nontreatment contrast	CG: not receiving any treatment; TG: acupuncture [40]	To assess the effects of acupuncture	Observe the progression of the condition and patient recovery	No blinding; placebo effects of acupuncture are not eliminated. This treatment would be contrary to medical ethics when treatment involves acute or severe conditions
(2)	Complementary contrast	CG: standard western medicine (with [41] or without [42] placebo acupuncture); TG: standard western medicine and acupuncture	To compare the effects of western medicine and western medicine plus acupuncture	Clarify the costs and side effects of acupuncture as the complementary therapy	Blinding is inadequate unless placebo acupuncture is used. Comparisons with standard treatments cannot be made, and the *β* error may not be correct
(3)	Alternative contrast	CG: standard western medicine; TG: acupuncture [43]	To compare the effects of acupuncture and western medicine	Demonstrate the effectiveness of acupuncture as the alternative therapy and assess the costs and side effects	No blinding; placebo effects of acupuncture are not eliminated, resulting in the *β* error
(4)	Nonacupuncture contrast	CG: nonpenetrating intervention, for example, TENS or laser acupuncture; TG: acupuncture [44, 45]	To assess the different effects of acupuncture, TENS, and laser acupuncture	Similar amounts of time and attention are spent on each group, thereby helping to eliminate some of the placebo effect	It is impossible to ensure blinding because of the substantial differences between the CG intervention and acupuncture. The CG intervention may have therapeutic effects. The effects of acupuncture are underestimated
(5)	No effects of nonacupuncture contrast	CG: mock nonpenetrating intervention, for example mock-TENS or mock laser acupuncture; TG: acupuncture [46]	To observe whether the therapeutic effects of acupuncture are greater than those of a nonpenetrating placebo intervention	If the TG receives TENS or laser acupuncture, elimination of the placebo effect and blinding can be assured. The CG intervention has no therapeutic effects and allows the observation of the progression of the condition and patients' recovery	It is impossible to ensure blinding because of the substantial differences between the CG intervention and acupuncture
(6)	Sham acupuncture contrast	CG: insertion points are not acupoints or meridians; TG: acupuncture at acupoints [47, 48]	To compare the effects of acupoints with sham points	The needling methods are the same in the CG and TG, resulting in an optimal elimination of placebo effect, and blinding can be performed	The effects of acupuncture on pain are underestimated. Having no uniform protocol for the sham points precludes accurate comparison of the results and conditions. Participants with previous acupuncture experience may be conscious of the difference in acupoint site(s), which influences the blinding
(7)	Nonspecific sites contrast	CG: acupuncture at acupoints which are considered to produce no or only minor effects; TG: acupuncture at specific acupoints related to the objective illness [49, 50]	To compare the specific effects of acupuncture points	Needling methods are the same in the CG and TG, resulting in an optimal elimination of placebo effect, and blinding can be performed	The effects of acupuncture on pain are underestimated. Selecting an unsuitable acupoint in the CG would render the result invalid
(8)	Minimal acupuncture contrast^∗^	CG: insertion points are not acupoints, and needle penetration is to a depth of less than 4 mm; TG: standard acupuncture [48, 51]	To assess the effects of acupuncture at acupoints with manipulating stimulation	The elimination of placebo effect, blinding is ensured; the procedure is easily manipulated	Potential therapeutic effects in the control group. To increase the efficacy of blinding, acupuncturists decreased the manipulation in the TG which then reduced the effects and confounded the results, and analysis. “De-chi” was not attained
(9)	Superficial acupuncture contrast	CG: dull needles or other tools (e.g., needle tube, toothpicks) are slapped on acupoints and tapped on them; dull needles do not prick the skin; TG: standard acupuncture [52]	To examine whether acupuncture is more effective than placebo acupuncture	The physiological reaction in the control group is minimal; the procedure is easily manipulated and suitably applied without requiring a novel study design; the blinding is effective	The operating locations were restricted to areas patients could not see, such as the neck, upper back, and dorsal side of limbs. The sensation was minimal and “de-chi” was not attained, which influenced the blinding for patients with previous experience of acupuncture; thus, the protocol cannot be used in long-term research
(10)	Placebo needle contrast	CG: Streitberger's needle, Park Sham Device, or Takakura's needle with a blunt tip was tapped onto the skin; TG: Streitberger's needle, Park Sham Device, or Takakura's needle with a real needle [17–20]	To observe whether real acupuncture is more effective than placebo acupuncture	The placebo effect and blinding are regarded as optimal, and, thus, the protocol has been widely used with good confidence	The major limitation of placebo needles is the associated lack of the “de-chi” sensation. Certain body sites cannot undergo acupuncture with these devices, such as the fingers, toes, and scalp, as well as sites that require transverse insertion or oblique insertion. These devices do not overcome the problem of double-blinding [33]. The needling methods in the TG had to be limited, thereby reducing the effects; thus, this procedure cannot be used in long-term research
(11)	Combined multiple methods contrast				
	(a)	CG: medicine and acupuncture placebo; TG: acupuncture and placebo medicine [53]	To reduce the psychological influence	The blinding and elimination of placebo effects were enforced to contrast the specific therapeutic effects between acupuncture and medicine	The procedure is difficult to use in long-term research
	(b)	CG: placebo needles inserted into acupoints and real needles inserted into sham points; TG: real needles inserted into acupoints and placebo needles inserted into sham points [54]	To produce similar therapeutic experience in the 2 groups; promote blinding and eliminate placebo effects	Blinding was effective, and the nonspecific effect of placebo needles was reduced	Participants with previous acupuncture experience may be conscious of the difference in acupoint site(s), which affects the blinding

CG: Control group; TG: Test group; TENS: Transcutaneous electrical nerve stimulation; ^∗^Minimal acupuncture was termed superficial acupuncture in some studies [51].
